# HyperArc VMAT planning for single and multiple brain metastases stereotactic radiosurgery: a new treatment planning approach

**DOI:** 10.1186/s13014-017-0948-z

**Published:** 2018-01-29

**Authors:** Shingo Ohira, Yoshihiro Ueda, Yuichi Akino, Misaki Hashimoto, Akira Masaoka, Takero Hirata, Masayoshi Miyazaki, Masahiko Koizumi, Teruki Teshima

**Affiliations:** 1 Department of Radiation Oncology, Osaka International Cancer Institute, 3-1-69 Otemae, Chuo-ku, Osaka, 537-8567 Japan; 2 Department of Medical Physics and Engineering, Osaka University Graduate School of Medicine, 1-7 Yamadaoka, Suita, Osaka, 565-0871 Japan; 3 Division of Medical Physics, Oncology Center, Osaka University Hospital, 2-2 (D10) Yamadaoka, Suita, Osaka, 565-0871 Japan; 4 Department of Radiation Oncology, Yao Municipal Hospital, 1-3-1 Ryuge-cho, Yao, Osaka, 581-0069 Japan

**Keywords:** Brain metastases, Stereotactic radiosurgery, VMAT, HyperArc, Dosimetric parameter

## Abstract

**Purpose:**

The HyperArc VMAT (HA-VMAT) planning approach was newly developed to fulfill the demands of dose delivery for brain metastases stereotactic radiosurgery. We compared the dosimetric parameters of the HA-VMAT plan with those of the conventional VMAT (C-VMAT).

**Material and methods:**

For 23 patients (1–4 brain metastases), C-VMAT and HA-VMAT plans with a prescription dose of 20–24 Gy were retrospectively generated, and dosimetric parameters for PTV (homogeneity index, HI; conformity index, CI; gradient index, GI) and brain tissue (V_2Gy_-V_16Gy_) were evaluated. Subsequently, the physical characteristics (modulation complexity score for VMAT, MCSV; Monitor unit, MU) of both treatment approaches were compared.

**Results:**

HA-VMAT provided higher HI (1.41 ± 0.07 vs. 1.24 ± 0.07, *p* < 0.01), CI (0.93 ± 0.02 vs. 0.90 ± 0.05, *p* = 0.01) and lower GI (3.06 ± 0.42 vs. 3.91 ± 0.55, *p* < 0.01) values. Moderate-to-low dose spreads (V_4Gy_-V_16Gy_) were significantly reduced (*p* < 0.01) in the HA-VMAT plan over that of C-VMAT. HA-VMAT plans resulted in more complex MLC patterns (lower MCSV, *p* < 0.01) and higher MU (*p* < 0.01).

**Conclusions:**

HA-VMAT plans provided significantly higher conformity and rapid dose falloff with respect to the C-VMAT plans.

## Introduction

The incidence of brain metastases, which often cause neurological complications, is 20–40% in patients with cancer. Meanwhile, the cancer detection rate has begun to increase owing to improvements in systematic therapy for primary cancer, resulting in longer patient survival [1]. As regards radiotherapy for brain metastasis, a European Organization for Research and Treatment of Cancer 22,952–26,001 study has suggested that stereotactic radiosurgery (SRS), which delivers a high dose of radiation in a single fraction, is not inferior to surgical resection as regards local control rates [2]. Kondziolka et al. reported that the rate of local failure at 1 y was only 8% with an SRS boost added to whole brain radiotherapy (WBRT) but that this was 100% with WBRT alone [3]. The American Society for Radiation Oncology published guidelines for management for brain metastasis, in which SRS is considered as the primary selection to improve survival and may be the best treatment choice for multiple brain metastases when quality of life is considered as the most important outcome [4].

In SRS, a rapid dose falloff from the surface of a target is required because the target is contained within normal brain tissue. Recently developed sophisticated patient immobilization devices, multileaf collimators (MLCs) and volumetric imaging techniques applied during treatment can provide highly conformal and precise dose delivery with the use of linear accelerators [5–7]. In this regard, Wolf et al. reported that the application of volumetric modulated arc therapy (VMAT), with continuously varying gantry speed, MLC positions, and dose rates during delivery, offers the advantages of short treatment time with lower number of monitor units (MU) and better conformity in comparison with conventional cone-based SRS [8]. Further, Hua et al. showed that VMAT with the use of non-coplanar beam orientations significantly reduced peripheral doses when compared with coplanar VMAT [9].

Recently, a new solution to fulfill the demands of SRS dose delivery has been developed by Varian Medical Systems. The new treatment planning system (TPS) incorporates several specialized functions for generating a -HyperArc VMAT (HA-VMAT) plan (not regulatory approved in Japan as of Dec. 2017) with a minimal workload including automated settings for the location of the isocenter, non-coplanar beam arrangement, and collimator angles. The use of the newest TPS affords the possibility of delivering a more conformal dose to the target while reducing doses to surrounding tissues as far as possible. However, few studies have thus far evaluated the dosimetric advantages of HA-VMAT.

The aim of this study is to compare the dosimetric parameters for target and normal tissue for the new HA-VMAT planning approach with the corresponding parameters for conventional VMAT (C-VMAT).

## Materials and methods

### Patients and simulation

This retrospective study included 23 patients (median (range) age 66 (23–78) years; 16 male and 7 female) with 1–4 brain metastases treated in a single fractionated SRS at our institution. The study was approved by our ethics committee with written informed consent provided by the patients. Twelve of these patients presented a solitary metastasis, five patients 2, five patients 3, and one patient 4 brain metastases. The patient details are listed in Table 1. In the Table 1, the positive value of the tumor position indicates that the tumor locates left, posterior or superior direction relative to the center of the skull. For computed tomography (CT) simulations (Light Speed 16 or Revolution HD, GE Medical Systems, Milwaukee, WI), the parameters for image acquisitions were: slice thickness of 1- or 1.25-mm, pixel matrix of 512 × 512 pixels, and field of view of 35-cm. The CT scans were loaded into a TPS (Eclipse, version 13.7, Varian Medical Systems, Palo Alto, CA). For gross tumor volume (GTV) delineation, a T1-weighted magnetic resonance imaging scan with contrast medium (gadolinium) was registered to the CT scans. A 2-mm margin was added to the GTV to create the clinical target volume (CTV). The planning target volume (PTV) was created by adding an isotropic margin of 1-mm to the CTV.

**Table 1:** Patient characteristics. The positive value of the tumor position indicates that the tumor locates left, posterior or superior direction relative to the center of the skull

Patient#	Tumor position (cm)			Target volume(cc)	Tumor diameter(cm)	Prescription dose(Gy)
	L-R	A-P	S-I			
Single isocentric VMAT						
1	−2.9	5.4	2.6	3.1	1.1	20
2	−2.8	2.1	4.1	2.0	0.9	24
3	−0.5	7.5	−7.4	4.5	1.3	24
4	3.7	0.7	4.7	1.8	0.8	24
5	−2.3	2.5	−0.3	1.9	0.7	24
	−1.4	1.1	0.9	1.6	0.6	
6	−1.1	2.0	−2.8	2.2	0.9	24
	−4.2	0.6	−3.0	2.7	1.0	
7	−3.6	−4.9	3.3	6.0	2.0	24
	−4.4	−1.5	4.4	1.3	1.1	
8	0.9	2.7	4.0	11.7	2.2	24
9	−5.7	1.1	1.1	1.5	0.7	24
10	−3.1	−1.2	4.4	6.3	1.6	24
11	0.5	1.4	−3.7	5.4	1.4	24
12	1.7	3.2	−5.5	2.9	1.2	24
13	−1.9	4.8	−4.6	3.9	1.3	24
14	−5.5	0.2	2.2	2.2	0.9	24
15	1.2	2.5	0.7	8.5	1.7	20
Multi isocentric VMAT						
16	−5.6	−1.5	1.3	0.6	0.4	20
	−2.9	3.3	4.7	0.5	0.4	
	−0.9	6.4	−3.9	0.7	0.4	
17	5.2	−1.0	2.3	15.6	2.4	24
	−0.1	0.4	2.7	1.6	0.7	
	1.8	1.2	−4.1	3.1	1.1	
18	−4.8	0.6	−1.0	1.0	0.6	20
	−2.3	5.1	−0.7	0.6	0.4	
	3.3	−3.0	2.9	1.5	0.8	
19	−3.9	1.3	−5.7	2.1	0.9	24
	3.4	1.5	4.6	1.1	0.6	
20	−3.8	−1.6	3.2	0.4	0.3	24
	−2.8	−1.2	4.3	1.0	0.6	
	1.0	5.8	2.3	2.7	1.1	
	−3.4	3.6	−5.4	1.0	0.6	
21	5.1	1.2	3.3	3.9	1.3	24
	−3.3	−2.2	4.4	1.1	0.6	
22	5.1	2.5	−2.3	3.9	1.2	20
	6.7	1.0	−1.7	1.2	0.7	
	−0.6	−5.6	3.6	1.2	0.7	
23	−2.4	3.1	1.3	0.9	0.5	20
	1.8	2.3	5.0	1.4	0.7	
	−0.2	−3.6	−1.0	3.0	1.0	

### Conventional VMAT planning

All treatment plans were designed based on a TrueBeam STX linear accelerator equipped with a 2.5-mm leaf-width MLC to deliver 20–24 Gy in a single fraction with flattening filter free beams with 6-MV photon beam energy at a maximum dose rate of 1400 MU per minute. The arrangement of the number of isocenters, beam angle, and couch rotation angle was manually selected depending on the size and location of the tumor. In case multiple targets are located far from each other (more than 5 cm), the isocenters were positioned in the center of each target, and a treatment plan was generated for each isocenter (multiple-isocentric C-VMAT cases, patient #16–23). The C-VMAT plan design was summarized in Table 2. The treatment plans were generated to cover 95% volume of the PTV with the prescription dose. A tuning ring layer was created around the PTV to control the expansion of the 50% isodose line of the prescription dose. To allow the inhomogeneous dose in the PTV, no dose constraint was set on the maximum dose in the PTV during the optimization process (photon optimization algorithm) with 1.25-mm optimization resolution. All doses were calculated by means of an analytic anisotropic algorithm (AAA) with heterogeneity correction on the 1.25-mm grid size.

**Table 2:** Planning design for conventional VMAT

Patient #	Num of isocenter	Num of arcs	Couch angle (arc length)
1	1	3	0 (360)
2	1	5	0 (360), 90 (180)
3	1	4	315 (90), 0 (180), 90 (90)
4	1	5	0 (180)
5	1	6	0 (360)
6	1	5	270 (180), 315 (180), 0 (360), 45 (180)
7	1	5	0 (195), 60 (180)
8	1	4	0 (360)
9	1	5	0 (90), 45 (180), 90 (180)
10	1	6	0 (180)
11	1	8	315 (140), 0 (360), 45 (150)
12	1	7	0 (360), 45 (120), 90 (120)
13	1	6	0 (360), 90 (180)
14	1	5	0 (180)
15	1	5	315 (180), 0 (230)
16	3	4	300 (90), 330 (90), 0 (90)
		4	0 (360), 90 (180)
		5	0 (360), 70 (180), 90 (180)
17	3	5	280 (180)
		6	315 (180),0 (360), 45 (180), 90 (180)
		5	0 (240)
18	3	4	15 (200)
		3	0 (360)
		4	0 (190)
19	2	6	340 (180), 0 (360), 10 (180)
		6	0 (360), 30 (180), 60 (180), 90 (180)
20	3	5	0 (180), 45 (180)
		4	0 (360)
		5	0 (360)
21	2	4	0 (180), 90 (180)
		4	270 (180), 0 (180)
22	2	7	350 (180)
		5	0 (180)
23	3	4	0 (180)
		4	0 (180)
		4	0 (180)

### HyperArc VMAT treatment planning

The same CT image and structure set used for C-VMAT planning were loaded to the newly developed prototype TPS (Eclipse, version 15.5, Varian Medical Systems). Treatment plans were designed for the same linear accelerator (2.5-mm leaf-width MLC) as that for the C-VMAT planning. In the HA-VMAT planning, the isocenter was positioned automatically based on the selected single and multiple target structures while that was determined manually in the C-VMAT planning. Based on these structures, the collimator angle and field size were optimized to reduce irradiation of organs at risk and/or the normal tissue. In addition, four arc fields, three of which were non-coplanar, were also automatically arranged: one full or half coplanar arc with couch rotation of 0° and three half non-coplanar arcs with couch rotations of 315°, 45°, and 90° (or 270°). We utilized the virtual dry run function to eliminate the arc field among the four arcs that exhibits a potential collision between the gantry and couch when the patient is treated. An SRS normal tissue objective (NTO) was used in the optimization process, which was designed to generate treatment plans that feature steep dose decay in space from target-specific dose levels to low asymptotic dose levels. The SRS NTO automatically recognizes spatial arrangements of targets for which dose bridging between targets is likely to occur and attempts to prevent dose bridging from occurring at least at dose levels higher than 17% of the prescription. Dose constraint was not set on the maximum dose. The aperture shape controller (ASC), which increases the field size and decreases the complexity of the MLC aperture on the average was not used in this study. The prescription dose (20–24 Gy), photon beam energy (filter free beams with 6-MV), optimization resolution (1.25-mm), dose calculation algorithm (AAA) and grid size (1.25-mm) were same as the corresponding ones in the C-VMAT planning.

### Data analysis

The treatment plans were evaluated by the comparison of dosimetric parameters derived from the dose-volume histograms for metastatic tumors and for normal brain tissues. The homogeneity index (HI) was defined as follows: HI = D_max_/D_prescribed_, where D_max_ and D_prescribed_ denote the maximum and prescribed doses, respectively [10]. The conformity index (CI) represents the objective measure of how well the distribution of radiation follows the shape of the radiosurgical target: CI = (TV_PV_ × TV_PV_)/(TV × PV), where TV_PV_, TV, and PV represent the volume of the target covered by the prescription dose, target volume, and prescription isodose volume, respectively [11]. The gradient index (GI), which is an evaluation of dose falloff, was calculated as: GI = PV_50%_/PV, where PV_50%_ denotes 50% of the prescription isodose volume. For brainstem, D_0.1cc_, which was the dose to 0.1 cc of the volume, was evaluated. For brain tissues excluding the PTVs, volumes receiving a specific dose in the range of 2–16 Gy (V_2Gy_-V_16Gy_) were evaluated. Moreover, to evaluate the low dose spread to brain tissue, subgroup analysis was performed by dividing the patient group into single- and multi-isocentric C-VMAT cases.

To evaluate the complexity of the MLC patterns, the modulation complexity score for VMAT (MCSV) for each plan was calculated with our in-house software (MATLAB R2016a; MathWorks, Natick, MA, USA) and the overall MCSV was defined as the mean of MCSV for each arc. The MCSV was calculated based on the leaf sequence variability (LSV) parameter and aperture area variability (AAV) [12]. The LSV was defined for each control point (CP) considering in each bank the differences in position between adjacent MLC leaves.

1 $$ {pos}_{\mathrm{max}}(CP)={\left(\max \left({pos}_{n\in N}\right)-\min \left({pos}_{n\in N}\right)\right)}_{leafbank}, $$

2 $$ {\mathrm{LSV}}_{\mathrm{cp}}={\left[\frac{\sum_{n=1}^{N-1}\left({\mathrm{pos}}_{\mathrm{max}}-\left|{\mathrm{pos}}_n-{\mathrm{pos}}_{n+1}\right|\right)}{\left(N-1\right)\times {\mathrm{pos}}_{\mathrm{max}}}\right]}_{\mathrm{leftbank}}\times {\left[\frac{\sum_{n=1}^{N-1}{\mathrm{pos}}_{\mathrm{max}}-\left|{\mathrm{pos}}_n-{\mathrm{pos}}_{n+1}\right|}{\left(N-1\right)\times {\mathrm{pos}}_{\mathrm{max}}}\right]}_{rightbank}, $$

where *N* and *pos* are the number of moving leaves inside the jaws and the coordinate of leaf position, respectively. The AAV is calculated as the area defined by apertures of opposing leaves in the single control point normalized to the maximum area in the arc, defined by the maximum apertures for each leaf pair over all CPs in the arc:

3 $$ {\mathrm{AAA}}_{\mathrm{cp}}=\frac{\sum_{a=1}^A\left({\left({\mathrm{pos}}_a\right)}_{\mathrm{leftbank}}-{\left({\mathrm{pos}}_a\right)}_{\mathrm{rightbank}}\right)}{\sum_{a=1}^A\left(\max {\left({\mathrm{pos}}_a\right)}_{\mathrm{leftbank}\widehat{\mathrm{I}}\mathrm{arc}}-{\left(\max \left({\mathrm{pos}}_a\right)\right)}_{\mathrm{rightbank}\widehat{\mathrm{I}}\mathrm{arc}}\right)}, $$

where *A* is the number of leaves in the arc. Finally, the MCSV is calculated using following formula:

4 $$ MCSV={\sum}_{i-1}^{i-1}\left[\frac{\left({AAV}_{cp_i}+{AAV}_{cp_{i+1}}\right)}{2}\times \frac{\left({LSV}_{cp_i}+{LSV}_{cp_{i+1}}\right)}{2}\times \frac{MU_{cp_{i,i+1}}}{MU_{arc}}\right], $$

where MU_CP*i*,*i* + 1_ indicates the number of MU delivered between two successive control points (namely, CP_*i*_ and CP_(*i* + 1)_). The value of the MCSV decreases as the modulation complexity increases. Thus, MCSV = 1 indicates that the plan is designed with a fixed rectangular aperture with no leaves moving during the arc. In addition, the number of MU used in each treatment plan was calculated as follows: MU = (total number of MU for patient)/(number of isocenters).

The paired Wilcoxon’ signed-rank test (SPSS, version 24; SPSSInc, Chicago, IL) was performed for the statistical measure of the difference in dosimetric parameters (C-VMAT vs. HA-VMAT). A *p*-value of <0.05 was considered to indicate statistical significance.

## Results

**Fig. 1:**
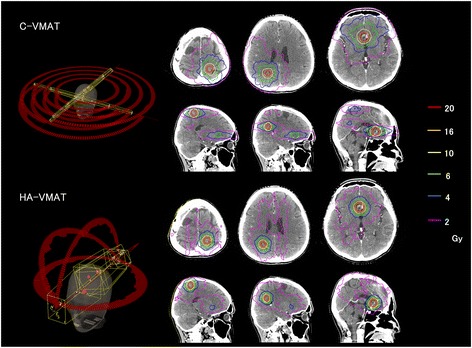
Comparison of beam arrangements and isodose distributions between conventional volumetric modulated arc therapy (C-VMAT) and HyperArc VMAT (HA-VMAT) plans for patient #23. The red, orange, yellow, green, blue, and purple lines indicate isodose lines of 20, 16, 10, 6, 4 and 2 Gy, respectively

Figure 1 compares the beam arrangements and isodose distributions between the C-VMAT and HA-VMAT plans for patient #23, who had 3 metastases. In this case, the C-VMAT plan located the isocenter for each center of tumor (three isocenters) while HA-VMAT plan used only one isocenter. It can be observed that the HA-VMAT plan provides a steeper dose gradient for PTV (for all targets) and the resultant absolute volumes of the brain tissue receiving 10 (50% isodose) and 6 Gy (30% isodose) are 4.6 and 17.4 cc lower than the corresponding ones of the C-VMAT plan. On the other hand, the HA-VMAT plan resulted in a higher absolute volume of 16.8 cc when receiving a very low dose (V_2Gy_).

**Fig. 2:**
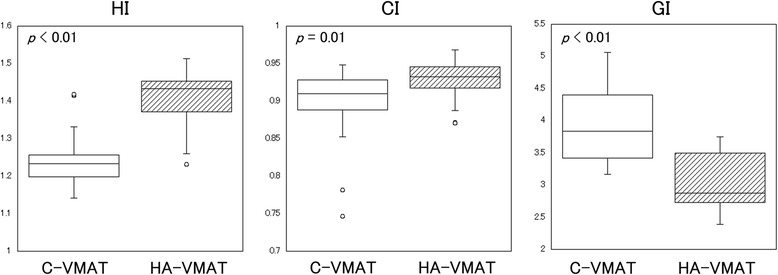
Boxplots of dosimetric parameters of homogeneity index (HI), conformity index (CI), and gradient index (GI) for conventional VMAT (C-VMAT) and HyperArc VMAT (HA-VMAT) plans. Boxes, median value and upper and lower quartiles; Whiskers, maximum and minimum values within 1.5 × inter-quartile range; Dots, outliers

Figure 2 shows the distributions of the dosimetric parameters with regard to HI, CI, and GI for both treatment approaches. The HA-VMAT plans achieve a significantly higher HI (mean ± standard deviation (SD); 1.24 ± 0.07 (C-VMAT) vs. 1.41 ± 0.07 (HA-VMAT), *p* < 0.01) and CI (0.90 ± 0.05 (C-VMAT) vs. 0.93 ± 0.02 (HA-VMAT), *p* = 0.01). In addition, the HA-VMAT plans generate a significant rapid dose falloff (GI) when compared with that of the C-VMAT plans (3.91 ± 0.55 (C-VMAT) vs. 3.06 ± 0.42 (HA-VMAT), *p* < 0.01).

The mean value (±SD) of D_0.1cc_ for brainstem was 3.36 (± 5.78) Gy and 3.34 (± 5.34) Gy for C-VMAT and HA-VMAT, respectively (*p* = 0.92). The absolute volume of the brain tissue receiving a specific dose is listed in Table 3 for both treatment approaches. In all cases (*n* = 23), the mean absolute volume was lower in the HA-VMAT plans, and a significant difference was observed at the dose level range from 16 to 4 Gy (V_16Gy_-V_4Gy_, *p* < 0.01). As opposed to the single-isocentric C-VMAT cases, HA-VMAT provided a significantly lower dose spread to the brain tissue at all evaluated dose levels (*p* < 0.01). In contrast, a very low dose volume (V_2Gy_) in HA-VMAT plan for multi-isocentric C-VMAT cases resulted in a somewhat larger dose spread (not significant, *p* > 0.05) than the C-VMAT plan.

**Table 3:** Dosimetric parameters for brain tissue for conventional volumetric modulated arc therapy (C-VMAT) and HyperArc VMAT (HA-VMAT) plans

Dosimetric parameter	All (*n* = 23)					Single-isocentric C-VMAT cases (*n* = 15)					Multi-isocentric C-VMAT cases (*n* = 8)				
	C-VMAT		HA-VMAT		*p*-value	C-VMAT		HA-VMAT		*p*-value	C-VMAT		HA-VMAT		*p*-value
	Mean	SD	Mean	SD		Mean	SD	Mean	SD		Mean	SD	Mean	SD	
V_16Gy_	5.3	3.8	3.8	2.8	<0.01	4.7	2.6	3.3	1.9	0.01	6.4	5.5	4.5	4.1	0.01
V_14Gy_	7.7	5.7	5.5	3.9	<0.01	6.8	3.9	4.8	2.7	0.01	9.5	8.1	6.7	5.6	0.01
V_12Gy_	11.3	8.4	7.9	5.6	<0.01	10.0	5.9	6.9	3.9	0.01	13.8	11.9	9.7	7.8	0.01
V_10Gy_	17.1	12.8	11.6	8.0	<0.01	15.3	9.5	10.2	5.8	0.01	20.5	17.7	14.3	11.2	0.01
V_8Gy_	27.0	20.6	17.9	12.4	<0.01	24.4	15.9	15.8	9.0	0.01	32.1	27.9	22.0	17.1	0.01
V_6Gy_	45.5	33.8	29.5	21.1	<0.01	41.0	28.0	25.5	14.6	0.01	53.8	43.7	37.0	29.5	0.01
V_4Gy_	86.9	62.3	58.1	48.0	<0.01	78.1	55.7	47.7	28.6	0.01	103.3	74.2	77.4	70.4	0.01
V_2Gy_	205.1	123.7	196.4	166.7	0.09	185.8	118.7	143.0	93.5	<0.01	241.2	132.8	296.4	228.7	0.09

**Fig. 3:**
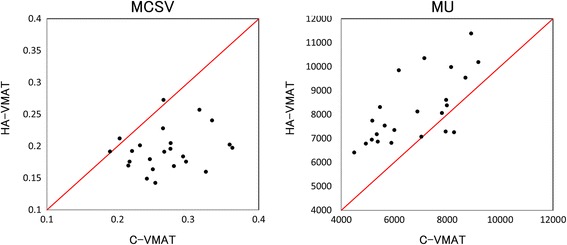
Distribution of physical characteristics of conventional volumetric modulated arc therapy (C-VMAT, horizontal axis) and HyperArc VMAT (HA-VMAT, vertical axis) for individual patients

The physical characteristics of the individual treatment plans between C-VMAT and HA-VMAT are directly compared in Fig. 3. Most of the HA-VMAT plans result in a lower value of the MCSV; in other words, HA-VMAT utilize smaller-sized segments to generate steeper dose gradients than C-VMAT. The mean (0.19 ± 0.03) of the MCSV in HA-VMAT was significantly lower (*p* < 0.01) than that of C-VMAT (0.27 ± 0.05). Consequently, HA-VMAT required significantly higher (*p* < 0.01) number of monitor units (8186 ± 1390 MU) than C-VMAT (6758 ± 1450 MU).

## Discussion

Historically, linear-accelerator-based SRS has been performed using the dynamic conformal arc radiotherapy technique with isocentric irradiation [7, 13]. Meanwhile, VMAT-based SRS treatment is a new area of active research. The VMAT technique in conjunction with the new generation of MLCs (2.5-mm leaf width) fulfills the demands of SRS dose delivery for small targets not only for a single target but also for multiple targets. In this regard, Thomas et al. have demonstrated that VMAT produces clinically equivalent conformity, dose falloff, 12-Gy isodose volume, low isodose spill, and reduced treatment time with respect to the corresponding parameters of a Gamma Knife unit, which has been the common technique for the treatment of multiple metastases [14]. Further, Garsa et al. reported that lower CI, and larger tumor volume were significant independent predictors of increase in tumor size after SRS for brain metastases [15]. Another investigation by Shiau et al. showed that longer interval of freedom before progression of the tumor after SRS for brain metastases was significantly associated with a higher prescription dose and a minimum dose ≥18 Gy was found to yield excellent local control [16]. However, particularly for large tumors, delivering a higher prescription dose can result in brain tissue receiving higher doses. Blonigen et al. showed that the dosimetric parameters corresponding to the range of V_8 Gy_-V_16 Gy_ were related with the incidence of brain radionecrosis when using linear-accelerator–based SRS [17]. In this study, we demonstrated that our HA-VMAT planning approach using the new generation of TPSs resulted in the significantly higher HI than C-VMAT plans in case the dose constraint for the maximum dose was not set in the optimization process. The higher HI plans are considered to be worse plan for conventional fractionated radiotherapy. Meanwhile, for hypo-fractionated radiotherapy such as SRS and SBRT, radiation oncologists sometimes allow the higher HI plans in case the maximum dose is located in the GTV. For example, Gamma Knife Unit commonly prescribed to the PTV with 50% isodose line, which means that the maximum dose within the target is twice as high as the prescription dose. Further improvement in the plan quality (higher CI, steeper dose falloff, and better sparing of brain tissue) was achieved in the HA-VMAT planning. Using this technique, dose escalation for relatively large tumors can be achieve while maintaining normal tissue tolerance. Several have investigators demonstrated the possibility of dose escalation for liver tumors, head-and-neck cancers, and glioblastoma multiforme using 4π radiotherapy, which utilizes the non-coplanar dose delivery technique developed by Dong et al. [18–20]. Further studies are expected to explore the possibility of dose escalation using HA-VMAT planning for brain metastases as well as other tumor sites.

Both 4π and HA-VMAT planning are non-coplanar planning platforms established on existing C-arm-type linear accelerators. The 4π optimization method begins with a candidate pool of 1162 beams evenly distributed throughout the 4π solid angle space with 6° of separation between adjacent beams [20]. Consequently, the machine has to “travel” between approximately 30 non-coplanar beams on their test plans. In contrast, HA-VMAT optimization creates the non-coplanar beams in conjunction with VMAT with four couch angles with one isocentric irradiation. For multiple targets, HA-VMAT can achieve faster dose delivery time than the 4π dose delivery technique. In HA-VMAT planning, the role of the SRS NTO is considered to be paramount, and the NTO is utilized to produce the most compact dose possible while also minimizing the dose between targets. Consequently, our results showed that the HA-VMAT plan utilized more complex MLC patterns with small segments (lower value of MCSV) than the C-VMAT plan. In this regard, Ohira et al. have reported that the mean MCSV value for intracranial tumors was 0.32 (range, 0.16–0.42) and VMAT plans with lower MCSV values resulted in less dosimetric accuracy, expressed as gamma pass rates [21]. Thus, careful dosimetric validation is imperative before introducing the HA-VMAT plan into clinical practice.

A concern about the use of the single-isocentric HA-VMAT for multiple targets is the increase of non-target tissue receiving a low dose. This is because two or more targets share the same MLC leaf pair, and the MLC will not have the ability to block radiation to the normal tissue around the multiple metastases [22]. Wu et al. reported the effect of using collimator optimization algorithm, which led to significant improvement in reducing the low dose to normal brain tissue, while retaining similar dose coverage to targets [23]. The collimator optimization algorithm for HA-VMAT planning was developed to solve this “island blocking” problem, and our results demonstrated that HA-VMAT affords lower volume receiving dose (≥ 4 Gy) for brain tissue over that for multi-isocentric irradiation. In contrast, V_2Gy_ of the HA-VMAT plan was higher, but this very low dose spread was not considered clinically significant because this dose was lower than the doses commonly delivered for whole brain irradiation in a single fraction.

Several limitations of this study warrant mention. First, our data could not support in-depth analysis of the effect of number of tumors (four tumors at maximum) and location of tumors on dosimetric parameters for HA-VMAT planning due to the limited number of patients. Second, the dosimetric accuracy of the HA-VMAT plan is not validated, and not all the available features for HA-VMAT treatment planning were used. The ASC can be selected five different level from “very low” to “very high”, and treatments with a high level of leaf modulation, such as head and neck treatments, is recommended a weight up to the “Moderate” setting. The ASC favors apertures of minimal local curvature by penalizing deviations from zero curvature as measured based on the positions of the tips of adjacent leaves that modulate the same spatially continuous target projection. The use of the function of ASC may increase the size of apertures and simplify MLC patterns. Third, the dosimetric parameters of the HA-VMAT plan were compared only with those of the C-VMAT plan. Here, we remark on the automated brain metastases treatment planning software, named Elements; Gevaert et al. have demonstrated the superiority of Element over conventional dynamic conformal arc therapy [24]. Moreover, there is a new generation of treatment units such as Cyber Knife and Gamma Knife [25, 26]. The advantages and disadvantages of SRS dose delivery with HA-VMAT planning based on the C-arm-type linear accelerator need to be compared with these treatment planning approaches and/or treatment machines. Fourth, a comparison of treatment time is not investigated in this study although treatment time is important for the clinical practice. It is expected that single-isocentric HA-VMAT will provide faster treatment time than multi-isocentric C-VMAT irradiation. Finally, typically in our institution, SRS is not performed in case tumors are close to a critical organ such as brainstem. Thus, further investigation is expected whether HA-VMAT can decrease doses for OARs when tumors are close to a critical organ in fractionated SRT. Despite these limitations, our quantitative data provide an important contribution to the active area of study for delivering an adequate dose to brain metastases while minimizing the dose to brain tissue.

## Conclusions

Our results clearly demonstrated the superiority of HA-VMAT planning with regard to generating highly conformity (CI) and rapid dose falloff (GI), and the radiation necrosis indicator (V_8Gy_-V_16Gy_) was significantly reduced in comparison with that of the C-VMAT plans. The resultant maximum dose (HI) in the HA-VMAT was significantly higher than C-VMAT. HA-VMAT can form one of the new choices for SRS dose delivery for both single target and multiple targets.

## Abbreviations

AAV: Aperture area variability
CI: Conformity index
CP: Control point
CT: Computed tomography
CTV: Clinical target volume
C-VMAT: Conventional volumetric modulated arc therapy
D_0.1cc_: Dose to 0.1 cc of the volume
D_max_: Maximum dose
D_prescribed_: Prescribed dose
GI: Gradient index
GTV: Gross tumor volume
HA-VMAT: HyperArc volumetric modulated arc therapy
HI: Homogeneity index
LSV: Leaf sequence variability
MCSV: Modulation complexity score for volumetric modulated arc therapy
MLC: Multileaf collimator
MU: Monitor units
NTO: Normal tissue objective
PTV: Planning target volume
PV: Prescription isodose volume
SRS: Stereotactic radiosurgery
TPS: Treatment planning system
TV: Target volume
TV_PV_: Volume of the target covered by the prescription dose
VMAT: Volumetric modulated arc therapy
V_*x*Gy_: Absolute volume receiving *x* Gy for a given organ
WBRT: Whole brain radiotherapy
